# Over-Expression of Semaphorin4D, Hypoxia-Inducible Factor-1α and Vascular Endothelial Growth Factor Is Related to Poor Prognosis in Ovarian Epithelial Cancer

**DOI:** 10.3390/ijms131013264

**Published:** 2012-10-16

**Authors:** Ying Chen, Lei Zhang, Yi Pan, Xiubao Ren, Quan Hao

**Affiliations:** 1 Department of Gynecologic Oncology, Tianjin Medical University Cancer Institute and Hospital, Tianjin 300060, China; E-Mails: lychenying2004@hotmail.com (Y.C.); zhanglei008_008@hotmail.com (L.Z.); 2 Department of Pathology, Tianjin Medical University Cancer Institute and Hospital, Tianjin 300060, China; E-Mail: panyi@hotmail.com; 3 Department of Biotherapy, Tianjin Medical University Cancer Institute and Hospital, Tianjin 300060, China

**Keywords:** Semaphorin4D, HIF-1, VEGF, ovarian cancer, prognosis

## Abstract

Semaphorin4D (SEMA4D) has been regarded as an important protein in tumor angiogenesis, though originally identified in neurodevelopment. SEMA4D is extensively expressed in several malignant solid tumors. Nevertheless, the function and expression of SEMA4D in epithelial ovarian cancer (EOC) is as yet not well understood. The aim of this study was to investigate SEMA4D expression in EOC and evaluate its clinical–pathological and prognostic significance. Immunohistochemistry was used to analyze SEMA4D expression and tumor angiogenesis-related proteins (HIF-1α and VEGF) in tissues from 40 patients with normal ovarian epithelia and 124 EOC patients. SEMA4D was found to be expressed in 61.3% of the 124 EOC tissues, which was significantly higher than in the normal ovarian epithelia (*p* < 0.001). SEMA4D expression correlated with HIF-1α and VEGF closely (*ρ* = 0.349 and 0.263, *p* < 0.001). Positive SEMA4D staining was significantly higher in tissues from patients with low histological grade, FIGO stage III-IV, lymph node metastasis and residual disease ≥1 cm (*p <* 0.05). In the Cox proportional hazard mode, SEMA4D expression and histologic grade were independent indicators of overall survival (OS) and progress-free survival (PFS) for EOC patients. These findings suggest that the cooperation of SEMA4D, HIF-1α, and VEGF may indicate poor prognosis for patients with EOC, thereby demonstrating that SEMA4D and its role in angiogenesis in EOC warrants further study.

## 1. Introduction

Semaphorins are a large family of transmembrane or glycosylphosphatidylinositol-linked proteins, which are grouped into eight classes [1]. Initially, the function of semaphorins has been best described in the nervous system. Recently, studies proved semaphorins are important players during angiogenesis, as they regulate blood vessel growth and endothelial cell homing during vessel development [2]. Semaphorin4D (SEMA4D, also known as CD100), a member of the semaphorin IV subfamily, has been found to promote angiogenesis upon binding its receptor Plexin-B1 both *in vitro* and *in vivo* [3,4].

Angiogenesis is essential for the growth, invasion, and metastasis of tumors [5]. Hypoxia-inducible factor-1 (HIF-1) is a major regulator of cell adaptation to hypoxic stress and plays a critical role in tumorigenesis and angiogenesis [6]. HIF-1 is a heterodimer composed of two subunits: HIF-1α and HIF-1β. HIF-1α is the oxygen-regulated subunit that determines HIF-1 activity. Under hypoxic conditions, HIF-1 transcriptional activity increases rapidly due to HIF-1α protein over-expression [7]. HIF-1 binds to hypoxia response elements (HRE) in the promoter of target genes and activates their expression to mediate adaptive responses to decreased oxygen concentration, such as the formation of new blood vessels via proliferation and migration of endothelial cells toward the developing tumor [8]. This response is influenced by increased production of pro-angiogenic proteins such as vascular endothelial growth factor (VEGF). Moreover, like other pro-angiogenic factors, semaphorins may be regulated by changes in oxygen tension [9].

A recent study indicated that SEMA4D is induced by hypoxia in a HIF-1 dependent manner and influences tumor vascularity in head and neck squamous cell carcinoma (HNSCC) [10] in a manner analogous to VEGF in order to attract Plexin-B1-expressing endothelial cells into the tumor for the purpose of promoting growth and vascularity. It also demonstrated that SEMA4D cooperated with VEGF to promote angiogenesis [11]. Complementally, SEMA4D, which is under the control of the HIF-family of transcription factors, cooperates with VEGF to promote tumor growth and vascularity in oral squamous cell carcinoma [12].

Epithelial ovarian cancer (EOC) is the leading killer among all gynecological malignancies [13]. Tumor angiogenesis had been found to have important prognostic significance in advanced ovarian cancer [14]. However, the function and expression of SEMA4D in EOC is not very well understood. Here, we examine the expression of SEMA4D, HIF-1α and VEGF in normal and cancerous ovarian tissues and assess their association with the established clinicopathologic factors of the disease as well as patient prognosis.

## 2. Results

### 2.1. Higher Expression of SEMA4D, HIF-1α and VEGF in Ovarian Cancer than in Normal Ovarian Tissues

**Figure 1:**
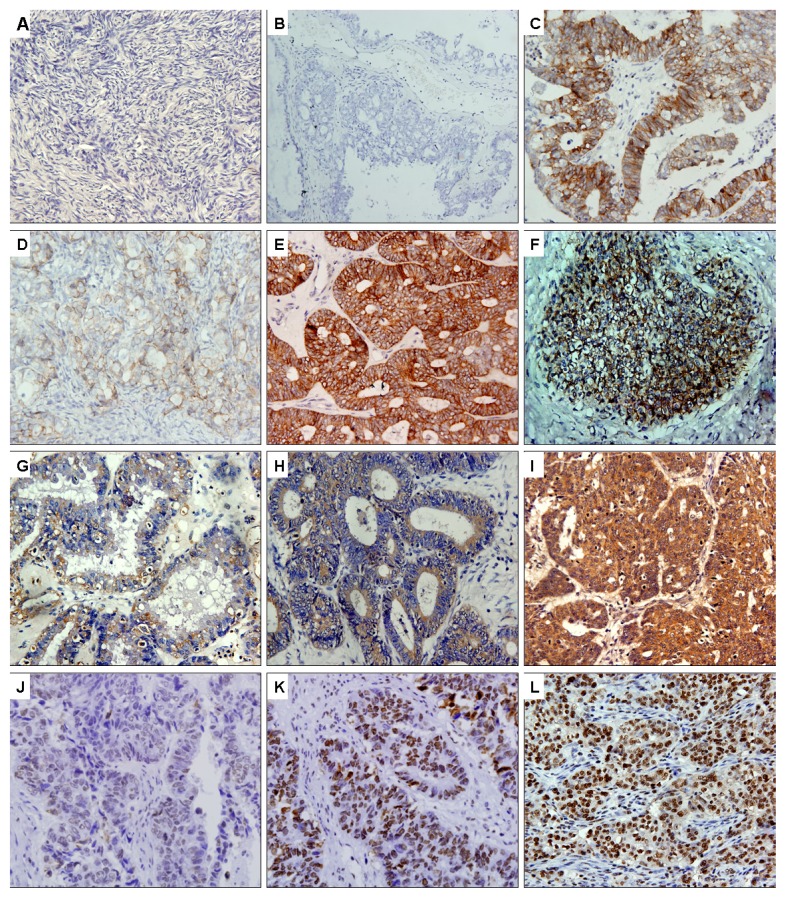
Representative images showing SEMA4D (**A**–**F**), VEGF (**G**–**I**) and HIF-1α (**J**–**L**) expressions. (**A**) SEMA4D negative expression in normal ovarian epithelia (400×); (**B**) mucinous ovarian adenocarcinoma (G2) with negative immunostaining of SEMA4D (200×); (**C**) serous ovarian adenocarcinoma (G2, FIGO Stage II) with immunostaining of SEMA4D in the membrane of tumor cells (400×; Evaluation: 5 points); (**D**) serous ovarian adenocarcinoma (G3, FIGO Stage I) with immunostaining of SEMA4D (400×; Evaluation: 3 points); (**E**) endometrioid ovarian adenocarcinoma (G2, FIGO Stage III) with immunostaining of SEMA4D (400×; Evaluation: 6 points); (**F**) ovarian adenocarcinoma (G3 to undifferentiated, FIGO Stage III) with immunostaining of SEMA4D (400×; Evaluation: 7 points); (**G**) serous ovarian adenocarcinoma (G2, FIGO Stage I) with immunostaining of VEGF in the cytoplasm of tumor cells (400×; Evaluation: 2 points); (**H**) endometrioid ovarian adenocarcinoma (G2, FIGO Stage II) with immunostaining of VEGF in the cytoplasm of tumor cells (400×; Evaluation: 4 points); (**I**) endometrioid ovarian adenocarcinoma (G3, FIGO Stage IV) with immunostaining of VEGF (400×; Evaluation: 7 points); (**J**) serous ovarian adenocarcinoma (G2, FIGO Stage II) with immunostaining of HIF-1α in the nuclei of tumor cells (400×; Evaluation: 1 point); (**K**) serous ovarian adenocarcinoma (G2, FIGO Stage III) with immunostaining of HIF-1α (400×; Evaluation: 5 points); (**L**) ovarian adenocarcinoma (G3 to undifferentiated, FIGO Stage III) with immunostaining of HIF-1α (400×; Evaluation: 7 points).

Immunohistochemistry revealed that 61.3% (76/124), 41.9% (52/124) and 48.4% (60/124) of ovarian cancer tissues stained positively for SEMA4D, VEGF and HIF-1α, which were significantly higher than the positive staining in normal ovarian epithelial tissues (61.3% *vs.* 10%, 41.9% *vs.* 10%, and 48.4% *vs.* 5%, *p* < 0.001, respectively; Table 1). Figure 1 shows the representative immunohistochemistry results.

### 2.2. Positive Correlation between the Expression of SEMA4D and HIF-1α or VEGF in Ovarian Cancer

Among 76 EOC tissues stained positively for SEMA4D, 52.6% (40/76) stained positively for VEGF and 63.2% (48/76) stained positively for HIF-1*α*. Likewise, among 60 EOC tissues stained positively for HIF-1α, 65.0% (39/60) stained positively for VEGF. The correlation of SEMA4D, VEGF and HIF-1α expression were closely (*ρ* = 0.263, 0.349 and 0.412, *p* < 0.05, respectively, see Table 2).

### 2.3. Expression of HIF-1α, VEGF and SEMA4D and Clinicopathologic Characteristics of EOC Patients

As shown in Table 3, over-expressions of SEMA4D and HIF-1α were closely related to EOC tissues with low histologic grade, advanced FIGO stage and lymph node (LN) metastasis (*p* < 0.05). Furthermore, over-expression of SEMA4D was also related to residual disease ≥1 cm. Over-expression of VEGF was closely related to EOC tissues with low histologic grade, advanced FIGO stage, chemotherapy resistance, and platinum resistant and refractory (*p* < 0.05). These results suggest that over-expression of HIF-1, VEGF and SEMA4D are associated with a more malignant ovarian cancer phenotype.

### 2.4. Survival Analysis of Prognosis Factors in EOC

**Figure 2:**
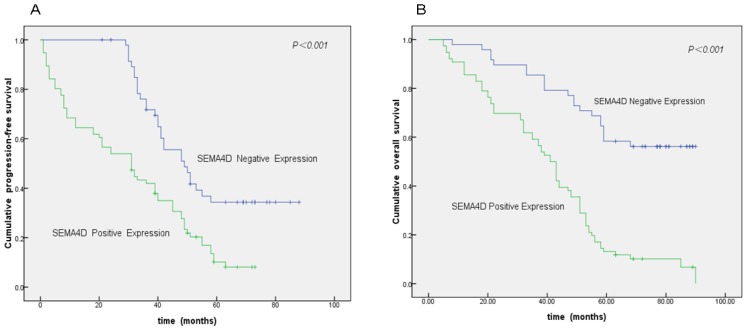
Kaplan-Meier analysis for the disease-free survival and overall survival of epithelial ovarian cancer patients according to SEMA4D expression in cancer cells. The survival curves were analyzed by the log rank test. (**A**) Epithelial ovarian cancer patients with positive SEMA4D expression showed shorter progress-free survival than those with negative expression; (**B**) Epithelial ovarian cancer patients with positive SEMA4D expression showed shorter overall survival than those with negative expression.

In univariate analysis, the media of OS and PFS were associated with the histologic grade, FIGO stage, LN metastasis, residual disease, tumors’ sensitivity to chemotherapy, response to chemotherapy, and expressions of HIF-1α and SEMA4D (*p* < 0.05, Table 4). Furthermore, as seen in Table 5, multivariate Cox analysis showed that advanced stage, low histologic grade and positive SEMA4D expression were the independent factors for evaluation of OS (*p* < 0.05). Additionally, low histologic grade and positive SEMA4D expression were the independent factors for evaluation of PFS (*p* < 0.05). Thus, patients with positive SEMA4D expression showed shorter OS and PFS than those with negative expression (Figure 2).

## 3. Discussion

There are striking similarities between the process of nerve growth and blood vessel branching [2]. SEMA4D, a protein originally shown to regulate axonal growth in the developing nervous system, has been identified as promoting angiogenesis in tumor progression.

Although SEMA4D has been previously explored in many types of cancer, including HNSCC, prostate cancer, colon cancer, breast cancer and lung cancer, its function remains unknown in ovarian cancer. In this study, data revealed SEMA4D positive expression in EOC tissues was obviously higher than in normal ovarian epithelial tissues.

The dimeric transcription factor HIF-1 is the key regulator of cellular response to hypoxia. HIF-1α is the regulatory subunit of HIF-1 and determines its activity. The HIF-1 heterodimer binds to HREs in the cis-acting sequences of target genes including VEGF, which is involved in adaptive pathways like angiogenesis [15]. VEGF, a pro-angiogenic factor whose expression increases upon cellular exposure to low oxygen tension, plays a crucial role in stimulating the switch from a hypoxic, avascular phenotype to a pro-angiogenic phenotype in growing tumors [16]. In the present study, we also found the positive expressions of VEGF and HIF-1α in EOC were significantly higher than in normal ovarian epithelial tissues.

Notably, previous studies had proved this angiogenic response elicited by SEMA4D was comparable to that by other well-known agiogenic molecules such as VEGF, HGF and bFGF and was dependent of up-regulation of these molecules [4,17]. HIF-1α accumulates in the cytoplasm and is translocated to the nucleus to perform transcriptional activity, which includes inducing expression of its downstream signaling molecule VEGF [18]. In addition, Zhou *et al*. [11] examined the contribution of SEMA4D to tumor-induced angiogenesis when compared to VEGF and suggested that targeting these proteins might represent a complementary or parallel mode of treatment for anti-angiogenic therapy of HNSCC and other solid tumors exhibiting insensitivity of anti-VEGF therapy. Moreover, our results showed that SEMA4D expression was positively correlated with HIF-1α and VEGF expression.

Furthermore, we found that over-expression of SEMA4D, VEGF and HIF-1α were all closely related to low histologic grade and advanced stage, while indicating a shorter OS, which was partially consistent with a previous study that reported HIF-1 and VEGF were associated with poor prognosis of ovarian cancer patients [19]. We therefore hypothesize that SEMA4D may be another pro-angiogenic factor in the downstream signaling pathway of HIF-1α, though further studies are needed to test this hypothesis.

The next univariated and multivariate survival analysis showed that low histologic grade and SEMA4D over-expression were the independent factors for predicting OS and PFS of EOC patients. Similarly, recent work has revealed a correlation between high levels of SEMA4D expression in some sarcomas and poor overall patient prognosis [20]. Therefore, we speculate that SEMA4D inhibition might offer a promising target for tumor anti-angiogenesis therapy and prevention of metastatic progression and invasion in EOC.

## 4. Experimental Section

### 4.1. Tissue Specimens

A total of 40 normal ovarian epithelia and 124 EOC tissues were obtained from patients (median age: 40 years and 50 years, respectively) admitted at the Department of Gynecologic Oncology, Tianjin Medical University Cancer Institute and Hospital from January 2004 to December 2006. Normal ovarian tissues came from women who underwent surgery for benign or malignant gynecological diseases other than ovarian carcinoma. Tumor specimens were collected from women during primary surgery and prior to the initiation of adjuvant therapy. Approval by the Institutional Review Board of Tianjin Medical University Cancer Institute and Hospital was obtained in advance, and the informed consent requirement was waived because the current study was performed as a retrospective review. Adjuvant chemotherapy consisted of paclitaxel (175 mg/m^2^) and carboplatin (6 AUC) and no patient was treated with an anti-angiogenic therapy. All patients were followed until death or the end of the follow-up period (March 31, 2012).

### 4.2. Immunohistochemical Staining

Sections of 4-mm thick serially cut from formalin-fixed and paraffin-embedded tissue blocks were deparaffinized, rehydrated and autoclave-treated at 121 °C for 10 min in 0.1 M citrate buffer (pH 6.0) to induce antigen retrieval. Endogenous peroxides in the section were blocked by incubation in 3% hydrogen peroxide for 5 min. Then, after being blocked with 0.5% goat serum for 60 min, the sections were incubated at 4 °C overnight with HIF-1α and VEGF primary antibodies (Santa Cruz Biotechnology Inc., Santa Cruz, CA, USA) or with SEMA4D (BD Biosciences, San Jose, CA, USA). The DAKO REAL EnVision Detection kit (DAKO) was subsequently applied for 30 min. Finally, sections were incubated in 3,3′-diaminobenzidine for 5 min, followed by Mayer’s hematoxylin counterstaining and mounting. Negative controls were obtained by replacing the primary antibody with isotype-matched monoclonal antibody. The percentage of positive cells was rated on the following point scale: no points (negative), ≤10% positive cells, regardless of staining intensity; 2 points, 11%–50% positive cells; 3 points, 51%–80% positive cells; and 4 points, ≥81% positive cells. The staining intensity was rated as follows: 1 point, weak intensity; 2 points, moderate intensity; and 3 points, strong intensity. Points for the percentage of positive cells and staining intensity were added, and specimens were attributed to two groups according to their overall score. Finally, specimens of ≤3 points were rated as negative, or as positive [21]. Two independent investigators blinded to clinical data performed the analysis.

### 4.3. Statistical Analysis

The spearman rank correlation was used to assess the degree of correlation among variables. The survival rate was determined by the Kaplan-Meier method, and the log rank test was used to determine significance. Factors that were deemed of potential importance by univariate analysis were included in the multivariate analysis. A result was considered significant when the *p* value was <0.05. All statistical analysis was performed with SPSS version 17.0 (SPSS Inc., Chicago, IL, USA).

## 5. Conclusions

In summary, we found SEMA4D expression was positively correlated with VEGF and HIF-1α in EOC and was a novel indicator of poor prognosis for EOC patients. We expect that SEMA4D may serve as a reliable tool for early and accurate prediction of tumor recurrence and may be a potential therapeutic target for EOC patients.

## Figures and Tables

**Table 1:** SEMA4D, VEGF and HIF-1α expressions in ovarian cancers and normal tissues.

Group	Cases (*N*)	SEMA4D positive expression *n* (%)	*p*	VEGF positive expression *n* (%)	*p*	HIF-1α positive expression *n* (%)	*p*
EOC tissues	124	76 (61.3)	<0.001	52 (41.9)	<0.001	60 (48.4)	<0.001
normal ovary	40	4 (10)		4 (10)		2 (5)	

**Table 2:** Relationship of HIF-1α, VEGF and SEMA4D expression in EOC.

		Cases (*N*)	VEGF expression *n* (%)		*ρ*	*p*	HIF-1α expression *n* (%)		*ρ*	*p*
								
			Negative	Positive	0.263	0.002	Negative	Positive	0.349	0.000
SEMA4D	Negative	48	36 (75.0)	12 (25.0)			36 (75.0)	12 (25.0)		
expression *n* (%)	Positive	76	36 (47.4)	40 (52.6)			28 (36.8)	48 (63.2)		
					0.412	0.000				
HIF-1α	Negative	64	51 (79.7)	13(20.3)						
expression *n* (%)	Positive	60	21 (35.0)	39 (65.0)						

**Table 3:** Correlation between HIF-1α, VEGF and SEMA4D expressions with clinicopathologic characteristics of EOC patients.

Variable	Cases (*N*)	SEMA4D positive expression		VEGF positive expression		HIF-1α positive expression	
				
		*n* (%)	*p*	*n* (%)	*p*	*n* (%)	*p*
Age			0.078		0.431		0.276
≤50 years	60	32 (53.3)		23 (38.3)		26 (43.3)	
>50 years	64	44 (68.8)		29 (45.3)		34 (53.1)	

Menopausal status			0.223		0.913		0.401
Yes	78	51 (65.4)		33 (42.3)		40 (51.3)	
No	46	25 (54.3)		19 (41.3)		20 (43.5)	

Pathologic type			0.709		0.581		0.390
serous carcinoma	80	50 (62.5)		35 (43.8)		41 (51.3)	
mucous and others	44	26 (59.1)		17 (38.6)		19 (43.2)	
Histologic grade			0.000		0.039		0.036
G_1–2_	49	20 (40.8)		15 (30.6)		18 (36.7)	
G_3_ or undifferentiated	75	56 (74.7)		37 (49.3)		42 (56.0)	

FIGO Stage			0.016		0.000		0.000
I–II	53	26 (49.1)		8 (15.1)		16 (30.2)	
III–IV	71	50 (70.4)		44 (62.0)		44 (62.0)	

LN metastasis			0.017		0.062		0.000
No	74	39 (52.7)		26 (35.1)		26 (35.1)	
Yes	50	37 (74.0)		26 (52.0)		34 (68.0)	

Residual disease			0.004		0.304		0.060
<1 cm	94	51 (54.3)		37 (39.4)		41 (43.6)	
≥1 cm	30	25 (83.3)		15 (50.0)		19 (63.3)	

Patients’ response to chemotherapy			0.349		0.010		0.108
CR	87	51 (58.6)		30 (34.5)		38 (43.7)	
PR, SD and PD	37	25 (67.6)		22 (59.5)		22 (59.5)	

Tumors’ sensitivity to chemotherapy			0.315		0.006		0.149
Platinum sensitive	92	54 (58.7)		32 (34.8)		41 (44.6)	
platinum resistant and refractory	32	22 (68.8)		20 (62.5)		19 (59.4)	

**Table 4:** Univariate analysis of OS and PFS in EOC patients.

Variable	Cases (*N*)	Media of OS	χ*^2^*	*p*	Media of PFS	χ*^2^*	*p*
Age			3.212	0.073		1.830	0.176
≤50 years	60	54			41		
>50 years	64	43			34		

Menopausal status			0.076	0.782		0.069	0.793
Yes	78	48					
No	46	51					

Pathologic type			0.354	0.552		0.002	0.967
serous carcinoma	80	43			41		
mucous and others	44	53			36		

Histologic grade			26.047	0.000		5.636	0.018
G_1–2_	49	85			48		
G_3_ or undifferentiated	75	43			33		

FIGO Stage			32.221	0.000		11.370	0.001
I–II	53	68			49		
III–IV	71	39			33		

LN metastasis			17.484	0.000		12.361	0.000
No	74	56			48		
Yes	50	35			33		

Residual disease			24.872	0.000		5.824	0.016
<1cm	94	54			41		
≥1cm	30	35			33		

Patients’ response to chemotherapy			16.060	0.000		5.393	0.020
CR	87	53			40		
PR, SD and PD	37	32			34		

Tumors’ sensitivity to chemotherapy			10.502	0.001		13.085	0.000
Platinum sensitive	92	53			45		
platinum resistant and refractory	32	22			10		

HIF-1α			25.811	0.000		4.639	0.031
Negative	64	59			41		
Positive	60	35			36		

VEGF			29.685	0.000		1.945	0.163
Negative	72	58			41		
Positive	52	32			34		

SEMA4D			34.933	0.000		16.541	0.000
Negative	48	NR			49		
Positive	76	41			31		

FIGO = International Federation of Gynecology and Obstetrics; NR = not reached; CR = complete response; PR = partial response; SD = stable disease; PD = progressive disease.

**Table 5:** Multivariate analysis with regard to OS and PFS.

	Overall survival				Disease-free survival			
		
			95.0% CI				95.0% CI	
						
Variable	*p*	HR	Lower	Upper	*p*	HR	Lower	Upper
VEGF	0.185	0.709	0.426	1.179				
Tumors’ sensitivity to chemotherapy	0.053	0.493	0.241	1.009	0.104	0.566	0.286	1.123
Patients’ response to chemotherapy	0.392	0.735	0.363	1.488	0.392	0.735	0.363	1.488
FIGO Stage	0.033	0.540	0.307	0.951	0.157	0.689	0.411	1.154
LN metastasis	0.793	1.068	0.654	1.742	0.793	1.068	0.654	1.742
Histologic grade	0.019	0.509	0.289	0.894	0.019	0.509	0.289	0.894
HIF-1α	0.113	0.671	0.409	1.099	0.113	0.671	0.409	1.099
SEMA4D	0.001	0.395	0.225	0.691	0.012	0.507	0.299	0.859
Residual disease	0.441	0.809	0.471	1.389	0.403	0.813	0.500	1.321
